# Supplementary material for: Identification of photoperiod-induced specific miRNAs in the adrenal glands of Sunite sheep (Ovis aries)
Source: Front Vet Sci. 2022 Jul 22;9:888207. doi: 10.3389/fvets.2022.888207 (PMC9354845; doi:10.3389/fvets.2022.888207)
Supplement: Supplementary file 1 [file Data_Sheet_1.ZIP › Supplementary file/miRDeep2 file.zip!/miRDeep2 file/Know_Structure/LP42A2/oar-mir-410.pdf]

miRBase precursor : oar-mir-410
Total read count : 921
oar-miR-410-5p read count : 5
oar-miR-410-3p read count : 912
remaining reads : 4

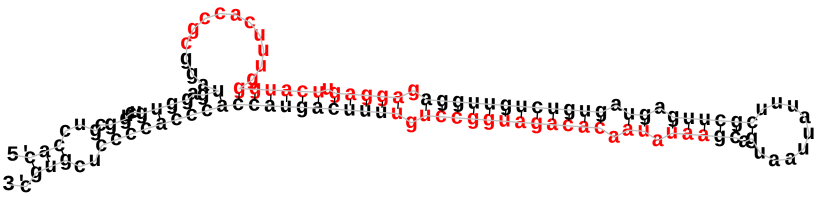

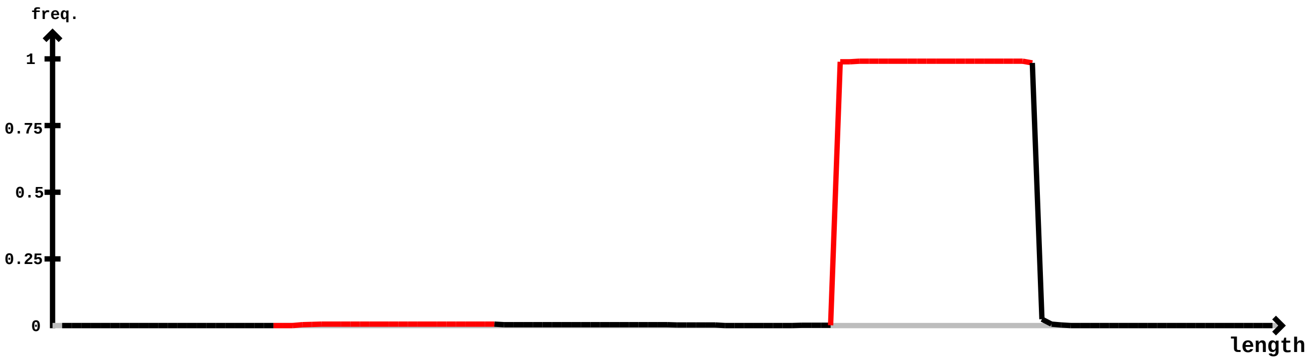


```
                                    oar-miR-410-5p
                                                                                           oar-miR-410-3p
5'- caccugcggucuguggaguaggcgccacuuuggguacuugaggagagguugucugugaugaguucgcuuuauuaaugacgaauauaacacagauggccuguuuucaguaccacccaccccucgugc   -3'    exp
    (((..(.(((..((((........))))....((((((.(((((.((((((((((((.((.((((((.........).))))).)).)))))))))))).)))))))))))....))).)..))).    reads      mm
    ........................ccacuuuggguacuugaggag.................................................................................    3          0
    .........................cacuuuggguacuugaggag.................................................................................    1          0
    ..........................acuuuggguacuugaggag.................................................................................    1          0
    .............................................agguugucugugaugagu...............................................................    1          0
    .............................................agguugucugugaugaguucgcu..........................................................    2          0
    ............................................................................gacgaauauaacacagauggccuguuu.......................    1          0
    ................................................................................aauauaacacagauggccug..........................    6          0
    ................................................................................aauauaacacagauggccugu.........................    885        0
    ................................................................................aauauaacacagauggccuguu........................    17         0
    ................................................................................aauauaacacagauggccuguuu.......................    1          0
    ................................................................................aauauaacacagauggccuguuuu......................    1          0
    ..................................................................................uauaacacagauggccuguuu.......................    1          0
    ..................................................................................uauaacacagauggccuguuuu......................    1          0
```
